# Supplementary material for: Communication, inclusion and psychological wellbeing among deaf and hard of hearing children: A qualitative study in the Gaza Strip
Source: PLOS Glob Public Health. 2023 Jun 6;3(6):e0001635. doi: 10.1371/journal.pgph.0001635 (PMC10243624; doi:10.1371/journal.pgph.0001635)
Supplement: S1 Appendix — (DOCX) [file pgph.0001635.s001.docx]

| **Variable** | **N** |
| --- | --- |
| ***Children and caregivers*** |  |
| Child interview | 17 |
| Caregiver interview | 10 |
| *Child age* |  |
| 6-9 | 15 |
| 10-12 | 12 |
| *Child sex* |  |
| Male | 14 |
| Female | 13 |
| *Caregiver sex* |  |
| Male | 2 |
| Female | 8 |
| *Severity of hearing loss* |  |
| Mild-moderate | 8 |
| Severe | 12 |
| Profound | 7 |
| *Assistive technology usage* |  |
| Hearing aid | 13 |
| Cochlear implant | 4 |
| *Communication mode* |  |
| Oral | 8 |
| Sign language | 13 |
| Bilingual | 6 |
| *Type of school* |  |
| Mainstream | 17 |
| Special | 10 |
| *Region* |  |
| Northern | 3 |
| Gaza City | 5 |
| Middle | 13 |
| Southern | 6 |
|  |  |
| ***Teachers*** |  |
| *Type of school* |  |
| Mainstream | 4 |
| Special | 4 |
| *Age of students* |  |
| 6-9 | 1 |
| 10-12 | 2 |
| All primary | 5 |
| *Severity of student hearing loss* |  |
| Mild-moderate | 5 |
| Severe-profound | 3 |
| *Region* |  |
| North | 1 |
| Gaza City | 2 |
| Middle | 2 |
| South | 3 |
|  |  |
| ***Focus group discussions*** |  |
| Mainstream education teachers | 6 |
| Special education teachers | 7 |
| School counsellors | 5 |
| MHPSS professionals | 2 |
| Deaf and hard of hearing adult and representatives of deaf and hard of hearing OPDs | 3 |
| Adults with other disability/impairment and representative of OPDs | 2 |
|  |  |

S1 Appendix. Participant characteristics
