# Supplementary material for: Communication, inclusion and psychological wellbeing among deaf and hard of hearing children: A qualitative study in the Gaza Strip
Source: PLOS Glob Public Health. 2023 Jun 6;3(6):e0001635. doi: 10.1371/journal.pgph.0001635 (PMC10243624; doi:10.1371/journal.pgph.0001635)
Supplement: S2 Appendix — (DOCX) [file pgph.0001635.s002.docx]

S2 Appendix. In-depth interview and focus group discussion topic guides

**Qualitative Interview Guide for
parents/caregivers of deaf and hard of hearing children in the Gaza Strip**

### Using the topic guides

These questions should be used to guide discussion but do not have to be used in the sequence listed below. The interviewer should follow up on any additional issues that may arise and seem important in relation to the issues above. The questions are written in full so that they are open, not leading and cover all the issues we need to explore. However, you can change the wording so that it’s more conversational, but make sure you cover the content and try not to lead the participant. We all have our own unconscious bias and assumptions and we need to ensure that these aren’t reflected in your questions.

**Materials needed:** information and consent sheet, voice recorder, spare batteries, notebook and pen, refreshments.

### Running the interviews

**Introduction:** Hi, my name is XXX. Thank you for speaking with me today. I’d like to talk to you about your child and supporting their wellbeing. We are talking to other parents, children and other teachers, so we can support the mental health and wellbeing of deaf and hard of hearing children.

Remind them of the issue of confidentiality and anonymity which is fully explained in the information and consent form that they completed. Reiterate that we will be asking questions about their experiences as a caregiver of a child with a disability. What you tell us about your experiences will be invaluable. If there are any questions you don’t want to answer any of them, they don’t have to – that’s ok. Check if they have any questions from the information and consent form about the research. Remind them that they are free to decline to answer any of the questions or stop the interview at any time.

1. **Background**

- Can you please tell me about your family?
  - Prompts: who lives in the house, number of children, who goes to work, who is the head of household
- What do you like to do together as a family?
- What is life like in the Gaza Strip?
  - Explore positives and challenges
- What are the services available to people living here?
  - Prompts: healthcare, school, work
  - Are you and others in the community able to access these services?

**2. Child**

- Can you tell me a little about your child’s hearing impairment
  - When did you first suspect they had difficulty hearing?
  - What did you do to find out?
  - How was the impairment caused?
  - At what age did she/he get diagnosed?
- What information did you receive on your child’s hearing impairment?
  - If no information received: what information would you like to have received?
- How is your child’s experience with day to day life and activities?
  - Explore positives and challenges
- How do you communicate with her/him?
- How do others in the family communicate with her/him?
- What is your child’s experience with family life?
  - Explore positives and challenges
  - Prompts: relationship with siblings, getting involved in decision making and family activities

**3. Healthcare**

- Have you sought healthcare services for her/his hearing impairment?
  - Who did you go to for this? What caused you to seek it?
  - What support was provided? How was the experience?
  - If not sought: has there been a time you needed a service but haven’t sought it? Explore why they didn’t and what support is needed
- What is your view on available hearing services in the Gaza Strip?
- What is you and your child’s experience of attending general healthcare services (e.g. primary healthcare)?
- How are you and your child typically treated by staff at healthcare facilities?

**4. School**

- Does your child go to a mainstream or special school?
- What does your child like about school?
- What does your child dislike about school?
- How is your child’s relationship with their peers?
  - How do you think the other children view your child’s hearing impairment?
- How easy is it for your child to make friends?
- What is your child’s experience with teachers at the school?
- How is your child’s experience of learning in school?
- Does your child receive any additional support at school to help them learn and join in activities?
  - Explore positives and challenges
  - Is there any support your child needs that is not available?

**5. Community**

- How do you think people in the community view people with disabilities?
- How do you think people in the community view people with a hearing impairment?
- How do you think other people in your community view your child’s hearing impairment?
- How do you think other people in your family view your child’s hearing impairment?
- Does your child get involved in community activities?
  - Prompts: Social events, clubs, festivals, religious events, community groups
- How do you think others in your community view you?

**6. Mental health and psychosocial support**

As you may know, many people across the world can develop poor mental health, and may feel unhappy, down, anxious or stressed.

- In some countries, it is not common to talk about mental health, whilst in others it is. How is it in your community?
- What do people in the community think about people with mental health issues?
- Is there anything at home, school or in the community that you think helps your child feel happy and good about themselves?
- Is there anything at home, school or in the community that you think helps your child feel sad and bad about themselves?
- How does your child cope with difficult situations or when feeling negative emotions, such as anger and sadness?
- Has your child ever exhibited symptoms of mental distress?
  - If yes: Why was this the case? Did they receive any support?
  - If they answer no: Could you see support being helpful for your child, if they needed it?
- In some countries, children take part in programmes to promote good mental health and wellbeing. This may include peer support groups, play groups, emotional training etc. Do you know of any such programmes? What does the programme provide?
- Do you think your child would like to take part in such a programme?
- Do you see benefit to your child taking part in such a programme?

Thank you for all the time and information you have given us today. Before we end, is there anything you would like to tell us or ask us?

**Qualitative Interview Guide for deaf and hard of hearing children in the Gaza Strip**

### Using the topic guides

These questions should be used to guide discussion but do not have to be used in the sequence listed below. The interviewer should follow up on any additional issues that may arise and seem important in relation to the issues above. The questions are written in full so that they are open, not leading and cover all the issues we need to explore. However, you can change the wording so that it’s more conversational, but make sure you cover the content and try not to lead the participant. We all have our own unconscious bias and assumptions and we need to ensure that these aren’t reflected in your questions.

**Introduction**

Hi, my name is XXX. Thank you for speaking with me today. I’d like to talk to you about your home, what you like to do for fun and a bit more about your life. We are talking to other children, family members and teachers, so we can support children with a hearing impairment in school. We want to help teachers better support you in school and make sure you are happy and feel good.

Remind them of the issue of confidentiality and anonymity which is fully explained in the information and consent form that they completed. Reiterate that we will be asking questions about their experiences as a caregiver of a child with a disability. What you tell us about your experiences will be invaluable. If there are any questions you don’t want to answer any of them, they don’t have to – that’s ok. Check if they have any questions from the information and consent form about the research. Remind them that they are free to decline to answer any of the questions or stop the interview at any time.

**1. Background**

- Can you please tell me about your family?
  - Who lives in your house?
  - How many brothers/sisters/cousins live with you?
- What do you like to do together as a family?
  - If the child has siblings
    - What do you like to do with your siblings?
    - How often do you get to do this together?
  - Ask about play – what do they find fun? Who do they like to play with?
- How do you communicate with your family?
  - If they answer sign language
    - How do you communicate with someone who does not know sign language?

**2. School**

- What school do you go to?
- Can you tell me what you enjoy about school?
- What is your least favourite thing about school?
- What are the children in your class like?
  - Do they talk to you about your deafness/hearing impairment?
- Are there other children with a hearing impairment in your class?
  - How does that make you feel?
- Tell me a bit about your friends at school
  - What do you like to do together?
  - How is it playing together at break times?
  - Do you have any difficulty playing together?
- What is it like making friends at school?
- Do you have both hearing and deaf friends?
  - How do you communicate with your hearing friends?
  - How do you communicate with your deaf friends?
- Do you think you are treated the same as all children at school?
- What do you think about the teachers at your school?
  - Who is your favourite teacher? Why are they your favourite?
  - Is there anything you don’t like about the teachers?
  - Do they talk to you about your deafness/hearing impairment?
- How do you communicate with the teachers?
- How do the teachers treat you?
  - Do you think this is the same as other children?
- How is it learning at school?
  - Explore positives and challenges
- Do you receive any additional support at school to help them learn and join in activities?
  - Explore positives and challenges
  - Is there any support you need that is not available?

**3. Mental health and psychosocial support**

- What makes you feel happy and good about yourself? (use emotions cards if needed)

Sometimes children can feel sad, unhappy or nervous. And it can happen for many reasons, like problems at home, or with friends, or maybe they have a problem they can’t solve by themselves. Imagine someone in your class was feeling like this.

- What makes you feel sad, unhappy or nervous? (use emotions cards)
- What do you do to feel better if you are feeling sad?
  - How does that make you feel?
- If you or someone in your class was feeling sad or stressed at school, what could teachers or friends do to make you/them feel better?
- What would help you to feel happy and confident at school?
  - What could teachers do to help you?

**4. Healthcare – may need to ask the parent**

- Can you tell me a little bit about your hearing impairment?
- Does anyone else in your family have a hearing impairment?
  - How does that make you feel?
- Have you been to visit a doctor for your deafness/hearing impairment?
  - What help did they give you?
- How often do you go to the doctor? (doesn’t need to be related to their hearing impairment only)
  - Prompt: once a week, once a month, once a year
- How do you feel when you go to the doctor?
  - Explore positives and challenges

**5. Community**

- How do you think your family view you?
  - Prompt: What do they think of your deafness/hearing impairment?
- How do you think your neighbours view you?
  - Prompt: What do they think of your deafness/hearing impairment?
- How do you find taking part in community activities with other children and groups?
  - Prompt: For example, festivals, religious events, sporting events

Thank you for all the time and information you have given us today. Before we end, is there anything you would like to tell us or ask us?

**Qualitative Interview Guide for
teachers of deaf and hard of hearing children in the Gaza Strip**

### Using the topic guides

These questions should be used to guide discussion but do not have to be used in the sequence listed below. The interviewer should follow up on any additional issues that may arise and seem important in relation to the issues above. The questions are written in full so that they are open, not leading and cover all the issues we need to explore. However, you can change the wording so that it’s more conversational, but make sure you cover the content and try not to lead the participant. We all have our own unconscious bias and assumptions and we need to ensure that these aren’t reflected in your questions.

**Materials needed:** information and consent sheet, voice recorder, spare batteries, notebook and pen, refreshments.

### Running the interviews

**Introduction:** Hi, my name is XXX. Thank you for speaking with me today. I’d like to talk to you about your school, the children you teach and supporting their wellbeing. We are talking to other parents, children and other teachers, so we can support the mental health and wellbeing of deaf and hard of hearing children.

Remind them of the issue of confidentiality and anonymity which is fully explained in the information and consent form that they completed. Reiterate that we will be asking questions about their experiences as a caregiver of a child with a disability. What you tell us about your experiences will be invaluable. If there are any questions you don’t want to answer any of them, they don’t have to – that’s ok. Check if they have any questions from the information and consent form about the research. Remind them that they are free to decline to answer any of the questions or stop the interview at any time.

**1. Background**

- How long have you been a teacher?
- What type of school do you work in?
- What subjects do you teach?
- How many children are in your class? What age are they?

**2. Teaching deaf and hard of hearing children**

- How many children do you teach with a hearing impairment?
- How do you communicate with children with a hearing impairment?
  - If sign language: did you receive support to learn sign language?
  - If they don’t use sign language, explore why
- In your view, how is the experience of deaf and hard of hearing students in school?
  - Explore positives and challenges
- How is the experience of teaching a deaf or hard of hearing child?
  - Explore positives and challenges
- What training/advice have you received to support deaf and hard of hearing children?
- Is there any additional educational support provided to deaf and hard of hearing children at your school?
  - If yes: What is provided? What is your experience providing this support? What helps you need to provide this support?
  - If no: Do you believe this support is needed? Why isn’t there any support provided? What would help this to be provided?
- What is the experience of deaf and hard of hearing children with their peers?
  - Explore positives and challenges
- Have deaf and hard of hearing children spoken to you and other teachers about their classmates?
- Have other children asked/spoken to you or other teachers about deaf and hard of hearing classmates?
- Have other teachers asked/spoken to you about deaf and hard of hearing children?

**3. Attitudes towards disability**

- How do you think people in the community view people with disabilities?
  - If negative: Why do you think that is?
- How do you think people in the community view deaf and hard of hearing people?
  - If negative: Why do you think that is?
- How do you think others in your community view teachers of deaf and hard of hearing children or those with another disability
  - If negative: Why do you think that is?

**4. Mental health and psychosocial support at school**

As you may know, many people across the world can develop poor mental health, and may feel unhappy, down, anxious or stressed.

- In some countries, it is not common to talk about mental health, whilst in others it is. How is it in your community?
- What do people in the community think about people with mental health issues?
  - If negative: Why do you think that is?
- What information are you given about mental health as a teacher?
- What mental health support is provided at your school for children?
  - How able are deaf and hard of hearing children able to receive this support?
- Have any of the children you teach ever demonstrated mental health issues?
  - If yes: How did you recognise this? What did you do to help?
- *Deaf and hard of hearing children are often at a greater risk of mental health issues.* Why do you think this may be the case?

Thank you for all the time and information you have given us today. Before we end, is there anything you would like to tell us or ask us?

**Topic guide for focus group discussion with teachers**

### Using the topic guides

These questions should be used to guide discussion but do not have to be used in the sequence listed below. The interviewer should follow up on any additional issues that may arise and seem important in relation to the issues above. The questions are written in full so that they are open, not leading and cover all the issues we need to explore. However, you can change the wording so that it’s more conversational, but make sure you cover the content and try not to lead the participant. We all have our own unconscious bias and assumptions and we need to ensure that these aren’t reflected in your questions.

**Materials needed:** information and consent sheet, voice recorder, spare batteries, notebook and pen, refreshments.

**Introduction:** Hi, my name is XXX. Thank you for speaking with me today. I’d like to talk to you about deaf and hard of hearing children that you teach. We are talking to parents, children and other teachers, so we can support the mental health and wellbeing of deaf and hard of hearing children.

Remind them of the issue of confidentiality and anonymity which is fully explained in the information and consent form that they completed. Reiterate that we will be asking questions about their experiences as a caregiver of a child with a disability. What you tell us about your experiences will be invaluable. If there are any questions you don’t want to answer any of them, they don’t have to – that’s ok. Check if they have any questions from the information and consent form about the research. Remind them that they are free to decline to answer any of the questions or stop the interview at any time.

1. **Informed consent**

- Individually, all participants read/are read the information sheet and sign/thumbprint the consent form
- Check if they have any questions about the research
- Remind them that they are free to decline to answer any of the questions or leave the discussion at any time
- Establish ground rules of respect and privacy

**2. Introduction**

- Ice-breaker: Everyone introduces themselves and says a little about their school/class

**3. Mental health in schools**

- What training or support is provided to teachers on hearing impairment?
- What training or support is provided to teachers on mental health?
  - And specifically regarding mental health for deaf and hard of hearing children?
- What mental health support is available for deaf and hard of hearing children in school?
  - What is the process of identifying a child?
  - What is the process of referral?
  - What support is available for a child with mental health issues?
- What do you think supports good mental health and wellbeing among deaf and hard of hearing children at school?
- What programmes are there to help promote good mental health in deaf and hard of hearing children? For example, communication training for teachers, training social skills, building resilience, education on emotional recognition

Thank you for all the time and information you have given us today. Before we end, is there anything you would like to tell us or ask us?

**Topic guide for focus group discussion with mental health and psychosocial support specialists**

### Using the topic guides

These questions should be used to guide discussion but do not have to be used in the sequence listed below. The interviewer should follow up on any additional issues that may arise and seem important in relation to the issues above. The questions are written in full so that they are open, not leading and cover all the issues we need to explore. However, you can change the wording so that it’s more conversational, but make sure you cover the content and try not to lead the participant. We all have our own unconscious bias and assumptions and we need to ensure that these aren’t reflected in your questions.

**Materials needed:** information and consent sheet, voice recorder, spare batteries, notebook and pen, refreshments.

**Introduction:** Hi, my name is XXX. Thank you for speaking with me today. I’d like to talk to you about mental health and psychosocial support for children and deaf and hard of hearing children, specifically. We are talking to parents, children and teachers, so we can support the mental health and wellbeing of deaf and hard of hearing children.

Remind them of the issue of confidentiality and anonymity which is fully explained in the information and consent form that they completed. Reiterate that we will be asking questions about their experiences as a caregiver of a child with a disability. What you tell us about your experiences will be invaluable. If there are any questions you don’t want to answer any of them, they don’t have to – that’s ok. Check if they have any questions from the information and consent form about the research. Remind them that they are free to decline to answer any of the questions or stop the interview at any time.

1. **Informed consent**

- Individually, all participants read/are read the information sheet and sign/thumbprint the consent form
- Check if they have any questions about the research
- Remind them that they are free to decline to answer any of the questions or leave the discussion at any time
- Establish ground rules of respect and privacy

**2. Introduction**

- Ice-breaker: Everyone introduces themselves, ask what the individual does for work (or would like to do if they are not currently working)

**3. Mental health in Gaza**

- How is mental health thought of in the Gaza Strip and Palestinian culture?
- What support is available for children with mental health issues in the Gaza Strip?
  - Who typically delivers mental health support and prevention/promotion programmes for children?
  - How would one typically access these services?
  - How long does it typically take to be referred and get an appointment?
  - How much does it tend to cost?
- Are there any prevention/wellbeing promotion programmes for children?
  - e.g. Building social skills, resilience etc.
- What are some of the challenges to providing this care?
- What are some of the challenges for children and families trying to access this care?
- What support is needed to help address these challenges?

**4. Mental health of children with a hearing impairment**

- Deaf and hard of hearing children are often at a higher risk of mental health issues. Why do you think that is?
- What do you think supports good mental health and wellbeing among deaf and hard of hearing children?
- Do you know of any services/programmes specifically for deaf and hard of hearing children?
- Does this support differ to that given to other children?
- What are the challenges to providing support to children with a hearing impairment?
- What helps to provide this support?

Thank you for all the time and information you have given us today. Before we end, is there anything you would like to tell us or ask us?

**Topic guide for focus group discussion with deaf and hard of hearing adults and members of organisations of persons with disabilities**

### Using the topic guides

These questions should be used to guide discussion but do not have to be used in the sequence listed below. The interviewer should follow up on any additional issues that may arise and seem important in relation to the issues above. The questions are written in full so that they are open, not leading and cover all the issues we need to explore. However, you can change the wording so that it’s more conversational, but make sure you cover the content and try not to lead the participant. We all have our own unconscious bias and assumptions and we need to ensure that these aren’t reflected in your questions.

**Materials needed:** information and consent sheet, voice recorder, spare batteries, notebook and pen, refreshments.

**Introduction:** Hi, my name is XXX. Thank you for speaking with me today. I’d like to talk to you about deaf and hard of hearing children. We are talking to parents, children and other teachers, so we can support the mental health and wellbeing of deaf and hard of hearing children.

Remind them of the issue of confidentiality and anonymity which is fully explained in the information and consent form that they completed. Reiterate that we will be asking questions about their experiences as a caregiver of a child with a disability. What you tell us about your experiences will be invaluable. If there are any questions you don’t want to answer any of them, they don’t have to – that’s ok. Check if they have any questions from the information and consent form about the research. Remind them that they are free to decline to answer any of the questions or stop the interview at any time.

1. **Informed consent**

- Individually, all participants read/are read the information sheet and sign/thumbprint the consent form
- Check if they have any questions about the research
- Remind them that they are free to decline to answer any of the questions or leave the discussion at any time
- Establish ground rules of respect, and privacy

**2. Introduction**

- Ice-breaker: everyone introduces themselves, ask what the individual does for work (or would like to do if they are not currently working)

**3. Disability in the Gaza Strip**

- What are some of the perceptions of disability amongst the local community?
- What are the experiences of people with disabilities in the Gaza Strip?
  - Explore positives and challenges
  - Are they included within community activities? Are they included at school?
- What about the experiences of deaf and hard of people?
  - Do deaf and hard of hearing children face any particular challenges?
- What support services are available for children with disabilities?
  - What supports families and children to access these services?
  - Do families and children have any challenges in accessing these?
- Do you think there should be any additional support available for these children?

**4. Mental health and psychosocial support for children with disabilities**

- Is it common to talk about mental health in the community?
- How is mental health thought of in the Gaza Strip and Palestinian culture?
- Children with disabilities are often at a higher risk of mental health issues. Why do you think that is?
- What are the risks to the mental health of deaf and hard of hearing children?
- What do you think supports good mental health and wellbeing among children with disabilities?
  - What supports good mental health of deaf and hard of hearing children specifically?
- Do you know of any mental health support provided to children with disabilities? What about for deaf and hard of hearing children?
  - What is the experience of deaf and hard of hearing children accessing these?
  - Is there any additional support needed?
  - If no mental health support is provided: What support do you think should be available to help promote the wellbeing of a deaf or hard of hearing child in their day to day life?

Thank you for all the time and information you have given us today. Before we end, is there anything you would like to tell us or ask us?
