# Supplementary material for: Communication, inclusion and psychological wellbeing among deaf and hard of hearing children: A qualitative study in the Gaza Strip
Source: PLOS Glob Public Health. 2023 Jun 6;3(6):e0001635. doi: 10.1371/journal.pgph.0001635 (PMC10243624; doi:10.1371/journal.pgph.0001635)
Supplement: Attachment — Submitted filename: PLOS Global Public Health_Gaza_Response to Reviewers.docx [file pgph.0001635.s004.docx]

| Comment | Response |
| --- | --- |
| REVIEWER 1 |  |
| This is a good qualitative study on communication, inclusion, and psychological wellbeing among deaf and hard of hearing children. There is a good discussion on what sort of factors contribute to the psychological wellbeing and inclusion of Deaf and hard of hearing (DHH) children. The topic is interesting, and it seems the authors have spent some good time conceptualizing it. In addition, the general overview, and application of the study and its relevance in social contexts is missing. The following can help improve the manuscript. | Thank you for your comments. They are much appreciated. We believe that the revisions we have made from your recommendations have strengthened the paper. |
| Abstract The last bit of the abstract can be improved to better state the recommendations and implications of the findings. | We have included some recommendations at the end of the abstract from line 47 to 50 |
| Introduction There is room for improvement in this section. 1. Authors first must state the broad theme or topic of the study. After introducing the broad theme/topic, its academic and practical significance (if applicable) must be discussed. You should present a convincing response to the question, "Why should anyone care about this article?" Author has already presented some data regarding this, but it is not enough evidence for justification of the study. 2. The author should indicates some gaps, inconsistencies in the literature that the current study has addressed and should explain the study’s main contribution. | Thank you for your comment. The main driving factor for this research is the lack of available evidence on the experiences of DHH children in the Gaza Strip and the need for this information. We have added information on this at lines 80, line 90, line 95, and line 98. |
| Materials and Methods The method used is appropriate for the type of study presented in the article. 1. How the sample size (35) has been determined is not described, author should provide some scholarly reference here. | At line 135, we have included additional information on developing the sample size, based on Patton’s maximum variation sampling, which is cited. |
| 2.It is mentioned that the interviews with the school students, caregiver and teacher were In-depth Interviews, and three Focus Group Discussions were held with DHH adults and disability leaders, mental health specialists. The author should explain explained why different interview techniques were used for different interviews. | Thank you for this suggestion. We have included a new paragraph at line 160, which we believe adds clarity as to the aims of each method. |
| Results and Discussion The results section gives an interesting description of a number of themes that have most likely had an important impact on the studies of the interviewed students. This section is well written. 1."Communication, inclusion and psychological wellbeing among deaf and hard of hearing children: A qualitative study in the Gaza Strip" According to the title of your study, psychological wellbeing among deaf and hard of hearing children is a key factor of this study. But I did not find enough writing or analysis about ‘psychological wellbeing’ in the result section. | Thank you for this comment. We have aimed to make the analysis on psychological wellbeing clearer throughout the results section. For example, at line 261, line 340, line 410, line 493 and line 510. |
| Conclusion I suggest the authors club the conclusion, limitations of the study, and future prospectus and elaborate it under "Conclusions or concluding remarks" as a heading | Thank you for this suggestion. We have not amended the discussion as you have suggested, as we believe that having a separate limitations section is important for transparency. The current structure is our preference for the article. The editor has confirmed that we can continue with this structure.  Recommendations for future research, policy and practice are weaved throughout the discussion and we have not included a separate section, so as not to make the manuscript overly long. The editor has confirmed that this acceptable. Example recommendations for future research include line 598 and line 750. Example recommendations for policy and practice include line 615, line 639, line 668, and line 722, amongst others. |
|  | Thank you again for your comments on this manuscript. We appreciate you taking the time to review the original manuscript and these amendments. As we hope you agree, the manuscript is now improved and we believe ready to publish. We hope you will join us in disseminating this publication. |

| REVIEWER 2 |  |
| --- | --- |
| This is a very important study, giving voice to deaf and hard of hearing (DHH) children, adults and their advocates in the Gaza strip. By analyzing a sufficient sample of interviews and focus groups, and nicely presenting the findings, the authors point at the difficult situation of this population in terms of language deprivation, stigma, and lack of basic services. They indicate needs and suggest solutions, making this study valuable for policy makers and anyone aiming at improving the situation for this population. I would also like to commend the authors for including DHH people from Gaza strip in the steering committee of the research, thereby strengthening its rigor. | Thank you for your comments. They are much appreciated. We believe that the amendments now made from your recommendations have strengthened the paper. |
| The introduction is well-written. Under 'Materials and Methods' (p. 4 line 84), please summarize the exact number of participants involved in the study at each category (interviews with children, caregivers, teachers, focus groups, and how many participants per each focus group). | Thank you. We have now included this from line 102. |
| Sentence 104 starting with 'Referral systems' needs further clarification | We have provided clarification at line 123. |
| Appendix S1: Please provide the following information about the DHH children: Usage of hearing aids or cochlear implants, the age of fitting/implantation (if known), and communication mode (oral, sign, bilingual). | Thank you for this suggestion. We have included this information in the Appendix S1. |
| 4. Data collection: Were the interviewers structured or half-structured? Please provide the full topic guides of the interviews and the focus groups, in the text or as an appendix. | The interviews were semi-structured. We have included this information at line 160. And as suggested, we have included the topic guides as a supplementary material. |
| 5. Data analyses: This section is relatively weak. Please provide a detailed explanation of the different stages of TA that you conducted according to Braun and Clarke, who, and how many people were involved at each stage. If more than one person was involved in determining codes/categorizations, at what stage were these analyses compared and discussed, and how were discussions solved? | Thank you for this comment. From line 194, we have included additional detail on the steps of thematic analysis, making clear the authors involved at each step. |
| Results and Discussion  1. The authors mentioned in the introduction the unique situation of children in conflict zones, such as suffering from multiple stressors and traumas. They also mentioned that the research was carried out during the COVID-19 pandemic. Yet, these topics were not discussed in the study itself. Were there any data stemming from the interviews which could inform us on the experience of coping with hearing loss in these contexts? Were participants asked about conflict/pandemic issues? If no data exists, a reflective notation may help the readers understand why certain topics were not discussed with participants or not raised by them. | This is an interesting point, thank you. We have included information at lines 176 and 742. |
| 2. My overall impression of the study is that it advocates for the use of sign language in DHH children, their families, teachers, and community. While this is a very important recommendation, another direction exists as well, which is improving the oral-communication services and devices provided to DHH children and adults. These may include intensive speech therapies for children using hearing aids or cochlear implants (without which they can hardly benefit from them), auditory specialists (e.g., who can fit CI's), systematic newborn hearing screening, providing and informing on accessibility devices such as FM systems and lived transcriptions. These can be emphasized in more details both at the 'assistive technology and communication' section (e.g., line 309 onwards), and at the discussion and recommendations. | Thank you for your comment. We have included a new paragraph at line 656. |
| 3. Maybe related to that, the authors mention in the discussion the relatively positive situation of children in special schools, who use sign language, are engaged with DHH peers and seem to be at lower risk for poor mental health. This is of course important to note, yet, it is also important to consider in the discussion the word-wide dispute on schooling segregation and the role that special schools may at times play in segregating children from society and limiting their opportunities. It seems that in the current context at Gaza strip, special schools provide opportunities that lack in mainstream schools (which is well described by the authors), also because they are private. Yet, it is important to bring about a wider perspective on schooling options, based on experiences in other countries. Thus, adapting recommendations that strive at ideally providing DHH with all options, including full integration within mainstream schools (when done well). | Thank you, this is a good point. We have included this information at line 681. |
| 4. Results line 353: Please mention the (apparently) lack of systematic governmental or organizational training and resources for ambulant teachers, who can instruct mainstream teachers about the needs of DHH children and provide DHH children with personal educational and other assistances (thereby supporting their inclusion in mainstream schools). | Thank you. We have included additional information at line 438 and we believe this information comes throughout the discussion. |
| 5. Line 405: When surveying detriment factors, emphasis needs to be given also to structural and material barriers (such as lack of governmental services), in addition to cultural and attitudinal ones. | Thank you for your comment. Respondents did not report to us substantial information on structural and material barriers. What information we were given is provided in the results, regarding audiology services, sign language services, schools, and government action. |
| Discussion line 598: Mental health services for DHH people would benefit also from recruiting signing mental health professionals, DHH professionals, and sign language interpreters specialized in mental health. | Thank you for this suggestion. We have included this information at line 729. |
| 7. Finally, in your suggestions please provide an informative review of existing interventions designed especially for DHH children (e.g., at schools, families). These can include also interventions applied in high-income countries | Thank you for your comment. We have previously conducted a systematic literature review of mental health interventions for deaf and hard of hearing children and we mention key relevant findings from this at line 725. We have also aimed to provide recommendations of other interventions, such as parent training (line 668) and stigma-related interventions (line 615). |
| 1. Page 1 line 47: Please change to 'moderate to severe or profound (depending on your source)', instead of 'complete' hearing impairment. | Complete is the terminology used in the paper cited, but we agree that profound is more appropriate and we have changed this accordingly. |
| 2. Line 58: The word 'are' is redundant. | Thank you, we have amended. |
| 3. Line 88: 'and caregivers' is redundant. | Thank you, we have amended. |
| 4. Line 124: The word 'to' seems incorrect here. | Thank you, we have amended. |
|  | Thank you again for your comments on this manuscript. We appreciate you taking the time to review the original manuscript and these amendments. As we hope you agree, the manuscript is now improved and we believe ready to publish. We hope you will join us in disseminating this publication. |
